# Rational design of small molecules able to inhibit α-synuclein amyloid aggregation for the treatment of Parkinson’s disease

**DOI:** 10.1080/14756366.2020.1816999

**Published:** 2020-09-14

**Authors:** Serena Vittorio, Ilenia Adornato, Rosaria Gitto, Samuel Peña-Díaz, Salvador Ventura, Laura De Luca

**Affiliations:** a Department of Chemical, Biological, Pharmaceutical and Environmental Sciences, University of Messina Viale Palatucci, Messina, Italy; b Institut de Biotecnologia i Biomedicina, Universitat Autonoma de Barcelona, Spain; c Departament de Bioquimica i Biologia Molecular, Universitat Autonoma de Barcelona, Spain; d ICREA, Passeig Lluis Companys 23, Barcelona, Spain

**Keywords:** Parkinson’s disease, alpha-synuclein, ligand-based pharmacophore, aggregation inhibitors, docking studies

## Abstract

Parkinson’s disease is one of the most common neurodegenerative disorders in elderly age. One of the mechanisms involved in the neurodegeneration appears related to the aggregation of the presynaptic protein alpha synuclein (α-syn) into toxic oligomers and fibrils. To date, no highly effective treatment is currently available; therefore, there is an increasing interest in the search of new therapeutic tools. The modulation of α-syn aggregation represents an emergent and promising disease-modifying strategy for reducing or blocking the neurodegenerative process. Herein, by combining *in silico* and *in vitro* screenings we initially identified 3-(cinnamylsulfanyl)-5-(4-pyridinyl)-1,2,4-triazol-4-amine (**3**) as α-syn aggregation inhibitor that was then considered a promising hit for the further design of a new series of small molecules. Therefore, we rationally designed new hit-derivatives that were synthesised and evaluated by biological assays. Lastly, the binding mode of the newer inhibitors was predicted by docking studies.

## Graphical Abstract

**Figure:**
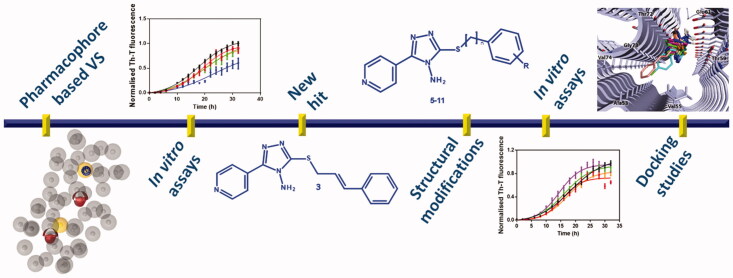

## 1. Introduction

Parkinson’s disease (PD) is a neurodegenerative disorder characterised by the loss of dopaminergic neurons in the *substantia nigra* of the brain. These cells are involved in the production of the neurotransmitter dopamine which regulates the muscular movements^1^. Typical manifestations of PD include motor symptoms due to the dopaminergic loss, like bradykinesia, rigidity, postural instability and rest tremor^2^. Additionally, non-motor features such as olfactory dysfunction, constipation, cognitive impairments, depression and sleep disorders can occur; these further symptoms are due to the implication of the neurodegenerative process in other areas of the peripheral and central nervous system^3^. The hallmark of PD is represented by the presence of neuronal inclusions, termed Lewis Bodies, mainly composed of aggregates of misfolded alpha synuclein (α-syn)^4^. α-Syn is a 140 aa presynaptic protein which regulates the release of neurotransmitters from the synaptic vesicles^5^. From a structural point of view, α-syn is organised in three different regions: the N-terminal domain (aa 1-60), the central NAC domain (aa 61-95) and the C-terminal domain (aa 96-140)^6^. In its monomeric soluble form, α-syn assumes an alpha helical conformation upon interaction with phospholipids,^7^ while in the pathological misfolded state, it aggregates into amyloid fibrils composed by parallel hydrogen bonded β-sheets^8^^,^^9^. The formation of these aggregates causes cytotoxicity through lipid membrane permeabilisation, mitochondrial damage and oxidative stress^10^. Another relevant mechanism that contributes to the propagation of neurodegeneration is the prion-like spread of α-syn aggregates. Indeed, experimental studies revealed that the injection of α-syn inclusions in animal’s brain promotes the formation of cellular inclusions at the injection site from where they can spread in other brain regions^11^. To date, the therapies available for the treatment of PD are addressed to reduce the motor symptoms and include the administration of drugs able to restore the level of dopamine. Among them the most used is L-Dopa, which acts as a prodrug being converted in dopamine in the brain^1^^,^^12^. Other dopaminergic drugs used for the treatment of PD are dopamine agonists like ropinirole or rotigotine, monoamine oxidase B (MAO-B) inhibitors such as rasagiline and selegiline and catechol-O-methyltransferase (COMT) inhibitors such as tolcapone and entacapone which inhibit the enzymes responsible of dopamine metabolism^2^^,^^13^. Unfortunately, the use of these drugs induces unwanted side effects such as dyskinesia, dizziness, headaches, nausea and somnolence^13^. More serious problems like hallucinations, confusion and impulse control disorders are often associated with the assumption of dopamine agonists^14^. Furthermore, these therapies lose their efficacy as the disease progresses and are unable to block or reduce the neurodegenerative process^15^^,^^16^. In the last decade, several efforts have been made to find a disease modifying strategy to halt or slow the neurodegeneration^17^. In this context, the inhibition of α-syn aggregation by small molecules proved to be a valid approach for the development of new therapeutics for the treatment of PD and several inhibitors have been discovered through high-throughput screening campaigns and drug repositioning^18^^,^^19^.

In this work, we applied a pharmacophore-based virtual screening approach as effective tool to discover novel α-syn aggregation inhibitors. Firstly, we developed a computational model that was subsequently combined with *in vitro* experiments to test their ability to reduce α-syn aggregation; as result we discovered a small molecule as promising inhibitor, which was used as lead compound for the development of a further series of compounds. Then, the designed molecules were synthesised, tested *in vitro* and studied to decipher the putative binding mode by molecular docking simulation.

## 2. Materials and Methods

### 2.1. Pharmacophore modelling and virtual screening

LigandScout V4.4^20^ was used for the pharmacophore generation and the virtual screening experiments. Three small molecules able to bind to the N-terminal region of α-syn have been selected from literature^21^ and used as training set. A shared-feature pharmacophore model was created applying the default settings.

All virtual screening runs were performed by setting the option “Get best matching conformation” as retrieval mode.

### 2.2. Chemistry

All reagents were used without further purification and bought from common commercial suppliers. Melting points were determined on a Buchi B-545 apparatus (BUCHILabortechnik AG Flawil, Switzerland). By combustion analysis (C, H, N) carried out on a Carlo Erba Model1106-Elemental Analyser we determined the purity of synthesised compounds; the results confirmed a ± 95% purity. Merck Silica Gel 60 F254 plates were used for analytical TLC (Merck KGaA, Darmstadt, Germany). For detection, iodine vapour and UV light (254 nm) were used. ^1^H and ^13^C NMR spectra were measured in dimethylsulfoxide-d6 (DMSO-d6) with a Varian Gemini 500 spectrometer (Varian Inc. Palo Alto, California USA); chemical shifts are expressed in δ (ppm).

#### 2.2.3. General procedure for the synthesis of pyridinyl-triazole derivatives 5-11

The 4-amino-5–(4-pyridinyl)-4H-1,2,4-triazole-3-thiol 12 (200 mg, 1,03 mmol) was dissolved in a mixture of NaOH (41 mg, 1,03 mmol) and MeOH (5 ml); after complete dissolution, the suitable benzyl bromide derivative (194,7 mg, 1,03 mmol) was added. The reaction mixture was stirred for 15 min at room temperature for desired compounds **5** and **7–11**, whereas for desired compound 6 the reaction time was 120 min. The resulting solid residue was washed with water, dried and recrystallized from ethanol to provide pure desired final pyridyl-triazole derivatives **5–11**. For all synthesised compounds the registered CAS numbers have been already assigned; However, the synthetic procedure, chemical and structural characterisation are not available in literature with exception of compound **5**, for which the structural characterisation is an agreement with literature^22^.

##### 2.2.3.1. 3-(Phenethylthio)-5-(pyridin-4-yl)-4H-1,2,4-triazol-4-amine (6)

CAS number: **691366-30-0**. Yield 12%. Yellow solid. M.p. 168–169 °C. ^1^H NMR (DMSO-d_6_): δ 3.04 (*t*, 2H, *J* = 7.74 Hz, CH_2_); 3.44 (*t*, 2H, *J* = 7.74 Hz, CH_2_); 6.19 (*s*, 2H, NH_2_); 7.21–8.72 (*m*, 9H, ArH) Anal. for C_15_H_15_N_5_S: C, 60.58%; H, 5.08%; N, 23.55%. Found C, 60.48%; H, 5.33%; N, 23.43%.

##### 2.2.3.2. 3-((4-Fluorobenzyl)thio)-5-(pyridin-4-yl)-4H-1,2,4-triazol-4-amine (7)

CAS number: **575460-68-**3. Yield 39%. Yellow solid. M.p. 186–187 °C. ^1^H NMR (DMSO-d_6_): δ 4.44 (*s*, 2H, CH_2_); 6.21 (*s*, 2H, NH_2_); 7.13–8.71 (*m*, 8H, ArH). ^13^C NMR (DMSO-d_6_): δ 34.0; 115.1 (d, ^2^*J_C-F_* = 19.1 Hz), 115.3 (d, ^2^*J_C-F_* = 19.1 Hz); 121.2; 121.3; 131.0 (d, ^3^*J_C-F_* = 8.6 Hz); 131.1 (d, ^3^*J_C-F_* = 8.6 Hz); 133.8 (d, ^4^*J_C-F_* = 2.9 Hz) 134.0; 150.1; 152.0; 154.5; 161.4 (d, ^1^*J_C-F_* = 243.2 Hz). Anal. for C_14_H_12_FN_5_S: C, 55.80%; H, 4.01%; N, 23.24%. Found C, 55.68%; H, 4.31%; N, 23.44%.

##### 2.2.3.3. 3-((3-Fluorobenzyl)thio)-5-(pyridin-4-yl)-4H-1,2,4-triazol–4-amine (8)

CAS number: **674812-86-3.** Yield 52%. Yellow solid. M.p. 164–165 °C. ^1^H NMR (DMSO-d6): δ 4.45 (*s*, 2H, CH_2_); 6.25 (*s*, 2H, NH_2_); 7.06–8.72 (*m*, 8H, ArH); ^13^C NMR (DMSO-d_6_): δ 34.6; 114.6 (d, ^2^*J_C-F_* = 20.9 Hz), 116.3 (d, ^2^*J_C-F_* = 21.0 Hz); 121.7; 121.8; 125.6 (d, ^4^*J_C-F_* = 2.7 Hz); 130.8 (d, ^3^*J_C-F_* = 8.2 Hz); 134.4, 141.0 (d, ^3^*J_C-F_* = 8.2 Hz); 150.5; 152.5; 154.8; 162.4 (d, ^1^*J_C-F_* = 243.4 Hz). Anal. for C_14_H_12_FN_5_S: C, 55.80%; H, 4.01%; N, 23.24%. Found C, 55.89%; H, 4.00%; N, 23.15%.

##### 2.2.3.4. 3-((4-Methylbenzyl)thio)-5-(pyridin-4-yl)-4H-1,2,4-triazol-4-amine (9)

CAS number: **676147-32-3.** Yield 13%. Yellow solid. M.p. 180–181 °C ^1^H NMR (DMSO-d_6_): δ 2.25 (*s*, 3H, CH_3_); 4.40 (*s*, 2H, CH_2_); 6.19 (*s*, 2H, NH_2_); 7.10–8.71 (*m*, 8H, ArH). ^13^C NMR (DMSO-d_6_): δ 21.0; 35.2; 110.0; 121.7; 121.8; 129.3; 129.6 134.4; 134.7; 137.1; 141.0; 150.4; 150.7; 152.4; 155.1. Anal. for C_15_H_15_N_5_S: C, 60.58%; H, 5.08%; N, 23.55%. Found C, 60.23%; H, 5.26%; N, 23.40%.

##### 2.2.3.5. 3-((3-Methylbenzyl)thio)-5-(pyridin-4-yl)-4H-1,2,4-triazol-4-amine (10)

CAS number: **676548-28-0.** Yield 16%. Whitish solid. M.p 175–176 °C; ^1^H NMR (DMSO-d_6_): δ 2.26 (*s*, 3H, CH_3_); 4.40 (*s*, 2H, CH_2_); 6.19 (*s*, 2H, NH_2_); 7.07–8.71 (*m*, 8H, ArH). ^13^C NMR (DMSO-d_6_): δ 21.3; 35.4; 121.7; 126.7; 128.5; 128.8; 130.0; 134.4; 137.6; 138.0; 150.6; 152.4; 155.1. Anal. for C_15_H_15_N_5_S: C, 60.58%; H, 5.08%; N, 23.55%. Found C, 60.70%; H, 5.01%; N, 23.32%.

##### 2.2.3.6. 3-((4-Chlorobenzyl)thio)-5-(pyridin-4-yl)-4H-1,2,4-triazol–4-amine (11)

CAS number: **901093-20-7**. Yield 39%. Yellow solid. M.p 198–199 °C; ^1^H NMR (DMSO-d_6_): δ 4.44 (*s*, 2H, CH_2_); 6.22 (*s*, 2H, NH_2_); 7.37–8.70 (*m*, 8H, ArH). ^13^C NMR (DMSO-d_6_): δ 34.4; 121.7; 121.8; 128.61; 129.0; 131.2; 131.5; 132.4; 134.4; 137.2; 150.4; 150.7; 152.5; 154.8. Anal. for C_14_H_12_ClN_5_S: C, 52.91%; H, 3.81%; N, 23.04%. Found C, 52.66%; H, 3.78%; N, 23.36%.

### 2.2. 2.3. Alpha synuclein aggregation and inhibition *in vitro* assays

WT α-Syn was expressed and purified as previously described^23^. The resultant protein was kept lyophilised at −80 °C. Lyophilised protein was delicately resuspended in sterile PBS 1X and filtered through 0.22-µm membrane to eliminate small aggregates. Aggregation assays were performed as previously described for ZPD-2 and SynuClean-D, the latter used as reference compound for kinetic studies^16^^,^^24^. Briefly, 70 µM of α-Syn, in a final volume of 150 µL, was placed in a sealed 96-well plate, which also contains 40 μM Th-T in PBS 1X, a 1/8” diameter Teflon polyball (*Polysciences Europe GmbH, Eppelheim, Germany*) and 100 μM of the different compounds or DMSO (in control samples). The plates were fixed in an orbital shaker Max-Q 4000 (*ThermoScientific, Waltham, Massachusetts, USA*) and incubated at 37 °C and 100 rpm. Th-T fluorescence emission was measured every 2 h in a Victor3.0 Multilabel Reader (*PerkinElmer, Waltham, Massachusetts, USA*) by exciting through a 430–450 nm filter and collecting with a 480-510 filter. Triplicates were performed and the kinetics were fitted with the following equation:

(1) $$\propto =1-\;\frac{1}{k_{b}(e^{k_{a}t}-1)\;+\;1}$$

In which k_b_ and k_a_ represent the homogeneous nucleation rate constant and the secondary rate constant (it means, fibril elongation and secondary nucleation), respectively^25^.

Light-scattering measurements were performed in a Cary Eclipse Fluorescence Spectrophotometer (Agilent, Santa Clara, California, USA). 80 µL of end-point aggregates was placed into a quartz cuvette and excited at 300 nm, the 90^°^ emission was thus collected between 280 and 360 nm.

For transmission electron microscopy (TEM) assays, endpoint treated and untreated aggregates diluted 1:10 in PBS 1X. Samples were then softly sonicated for 5 min and 5 µL of the resultant mixture were placed on a carbon-coated copper grid for 5 min. The grids were dried with filter paper to remove the excess and washed twice in miliQ water, whose excess was also removed. Finally, 5 μL of 2% (w/v) uranyl acetate solution was added and left incubate for 2 min. As previously, the excess of uranyl acetate was removed, and grids were left to air-dry for 10 min. Images were obtained using a Transmission Electron Microscopy Jeol 1400 (Peabody, Massachusetts, USA) operating at an accelerating voltage of 120 kV. A minimum of 30 fields were screened per sample, in order to collect representative images.

### 2.4. Docking studies

Docking studies were performed by Autodock4.2 software by using the solid-state NMR of alpha-synuclein fibrils retrieved from RCSB Protein Data Bank (PDB code 2N0A). The structure is characterised by a central part with a greek-key topology and terminal flexible loops. A grid box with dimension of 126 × 126 × 126 Å^3^ and centre *x* = 97.487, *y* = 148.695 and *z* = −34,111, was applied in order to include the aminoacid residues of the N-terminal region discarding the ones present in the unstructured flexible loops. Ligand structures were constructed by Vega ZZ software and energy minimised by following a conjugate gradient minimisation by AMMP calculation as implemented in VEGAZZ program^26^. The Lamarckian Genetic Algorithm was used to calculate 10 protein − ligand binding poses for each compound by using the default settings. The highest scored docking pose was chosen for analysis and representation. PyMOL software was used to visualise docking results while the analysis of the putative ligand–protein interactions was performed by Discovery Studio Visualiser^27^.

## 3. Results and discussion

### 3.1. Pharmacophore model generation, virtual screening and preliminary biological assay

With the aim to find new molecules able to inhibit α-syn aggregation, we used a computational approach to generate a ligand-based pharmacophore model by means of the software LigandScout^20^.

To build the model, we used a training set (TS) including α-syn aggregation inhibitors that target the N-terminal portion of the protein. Specifically, we selected three small molecules from literature^21^ belonging to different chemotypes as polyphenolic and non-polyphenolic inhibitors (Table 1) for which the binding interaction to N-terminal portion of α-syn is corroborated by experimental data.

**Table 1.**
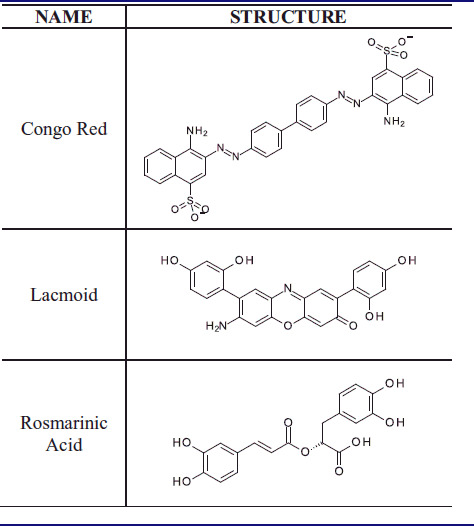
Chemical structures of the α-syn aggregation inhibitors used as training set (TS).

In particular, we considered only the common features for all the selected molecules of the TS for the model construction generating shared feature pharmacophore models.

**Figure 1.**
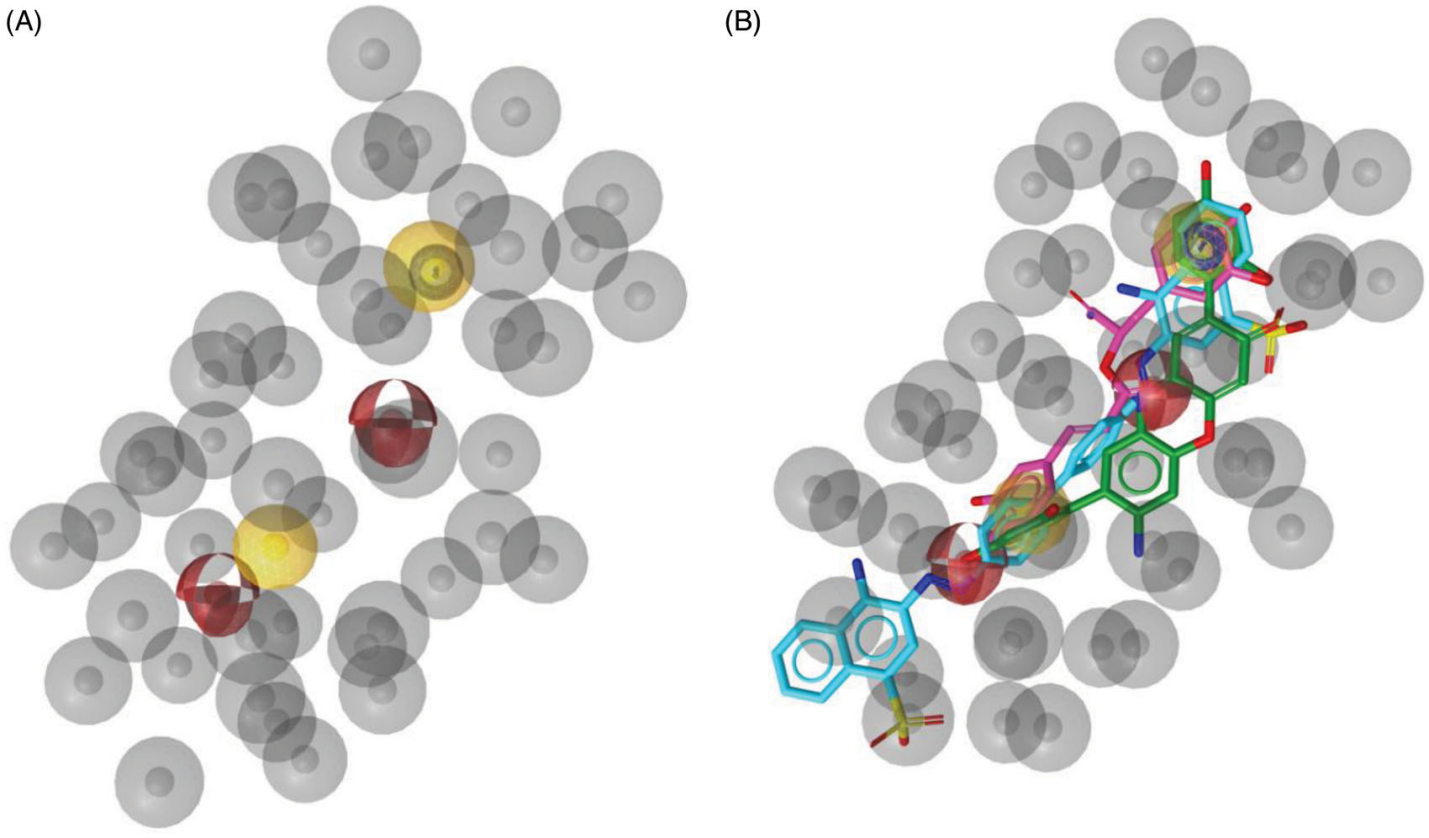
(A) Best scored ligand-based pharmacophore model constituted by two hydrogen bond acceptors (red spheres), two hydrophobic features (yellow spheres), one aromatic feature (blue circle). Forty-five excluded volumes are represented by grey spheres. (B) TS molecules aligned with the highest scored pharmacophore model.

As result, we obtained ten pharmacophore hypotheses and selected the hypothesis possessing the highest score to perform virtual screening studies. As displayed in Figure 1, the above-mentioned pharmacophore model consisted of five features: (i) two hydrogen bond acceptors, (ii) two hydrophobic features, (iii) one aromatic feature and 45 excluded volumes as forbidden areas.

**Figure 2.**
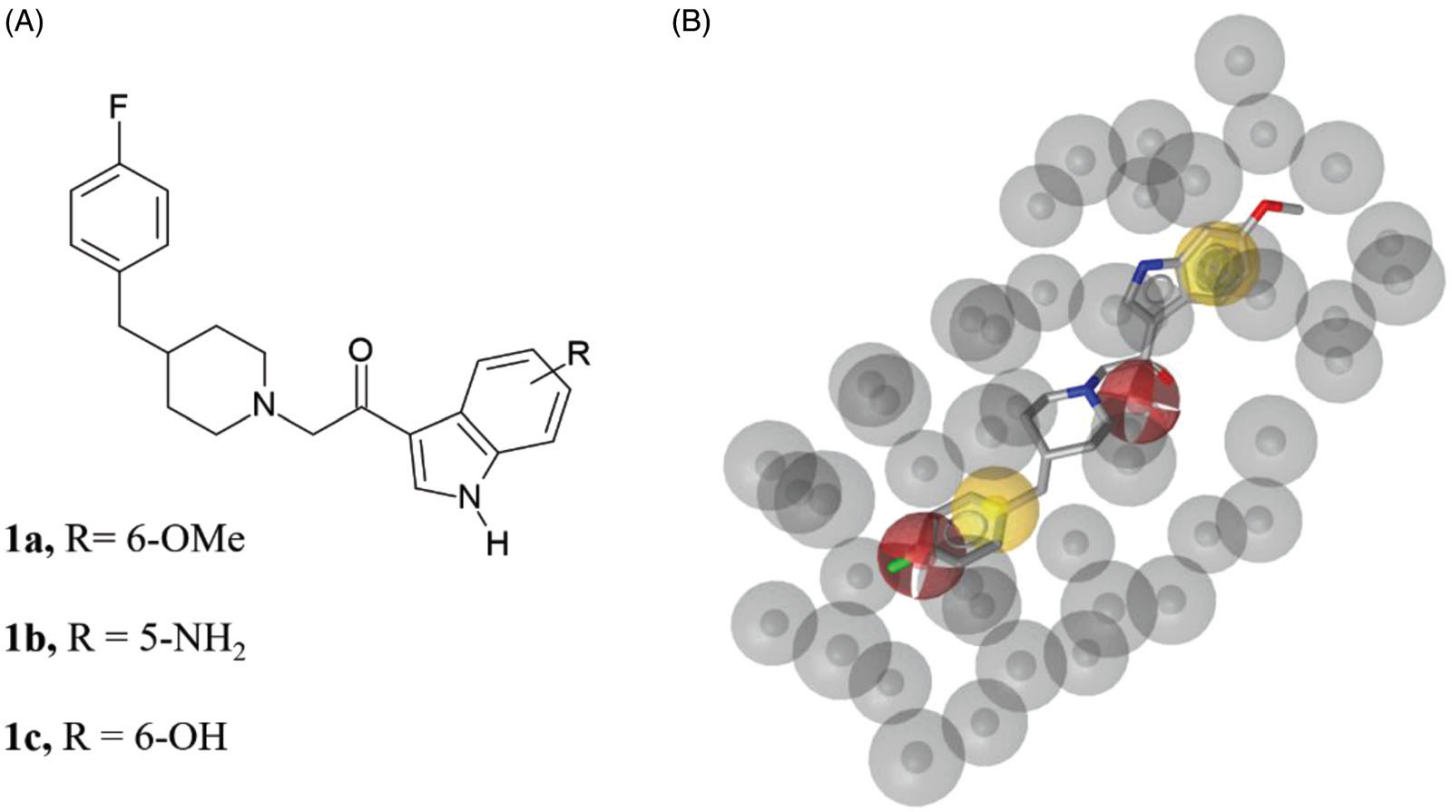
(A) Chemical structure of compounds **1a-c**. (B) Compound **1a** aligned to the pharmacophore model. Compound **1a** is represented by grey sticks.

The obtained pharmacophore model was used as a query to screen two three-dimensional databases containing small molecules belonging to different structural chemotypes: (i) the *in-house* 3 D database CHIME collecting 1329 molecules designed and synthesised by our group over the years, (ii) the MyriaScreen Diversity Library II, consisting of 10,000 compounds with druglike properties (https://www.sigmaaldrich.com/chemistry/chemistry-services/high-throughput-screening/screening-compounds.html). From CHIME, the virtual screening led to the identification of three hits **1a–c** (Figure 2(A)). Considering that these molecules shared a very similar structure, we decided to select only 2-[4-[(4-fluorophenyl)methyl]-1-piperidyl]-1-(6-methoxy-1H-indol-3-yl)ethenone (**1a**) displaying the highest pharmacophore fit-score as representative inhibitor filtered through virtual screening on CHIME database (Figure 2(B)).

**Figure 3.**
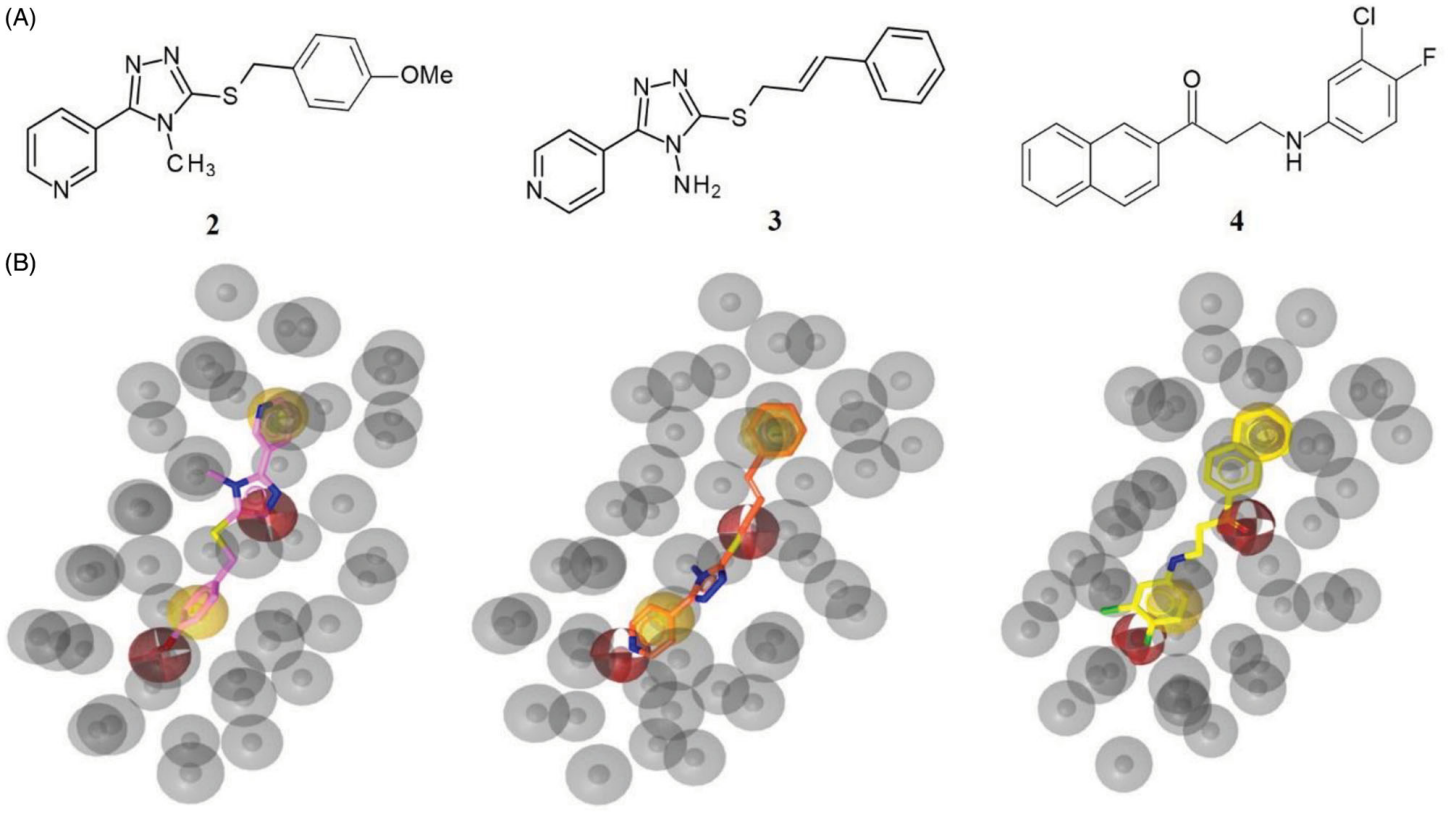
(A) Chemical structures of compounds **2**, **3** and **4**. (B) Alignment of each of the reported hits (represented by pink **(2)**, orange **(3)** and yellow **(4)** sticks) with the pharmacophore model.

The virtual screening on MyriaScreen Diversity Library II resulted in 113 hits (see supporting material). Among them, we selected three compounds (Figure 3.) such as 3-[5-[(4-methoxyphenyl)methylsulfanyl]-4-methyl-1,2,4-triazol-3-yl]pyridine (**2**), 3-(cinnamylsulfanyl)-5–(4-pyridinyl)-1,2,4-triazol-4-amine (**3**) and 3-(3-chloro-4-fluoro-anilino)-1-(2-naphthyl)propan-1-one (**4**); the selection of the compounds **2–4** was made basing on the pharmacophore-fit score value (>57) and the commercial availability from the supplier.

All selected compounds **1–4** proved to respect the Lipinski’s rule and no PAINS were *in silico* predicted by the online tool SwissAdme platform [http://www.swissadme.ch/]. To evaluate the ability to inhibit α-syn aggregation, we have got compounds **1–4** as follows. Compound **1** was readily synthesised in our laboratory following a previously optimised synthetic approach;^28^ while the three commercially available compounds **2–4** were purchased from Sigma Aldrich as supplier of bioactive molecules [https://www.sigmaaldrich.com/italy.html].

**Figure 4.**
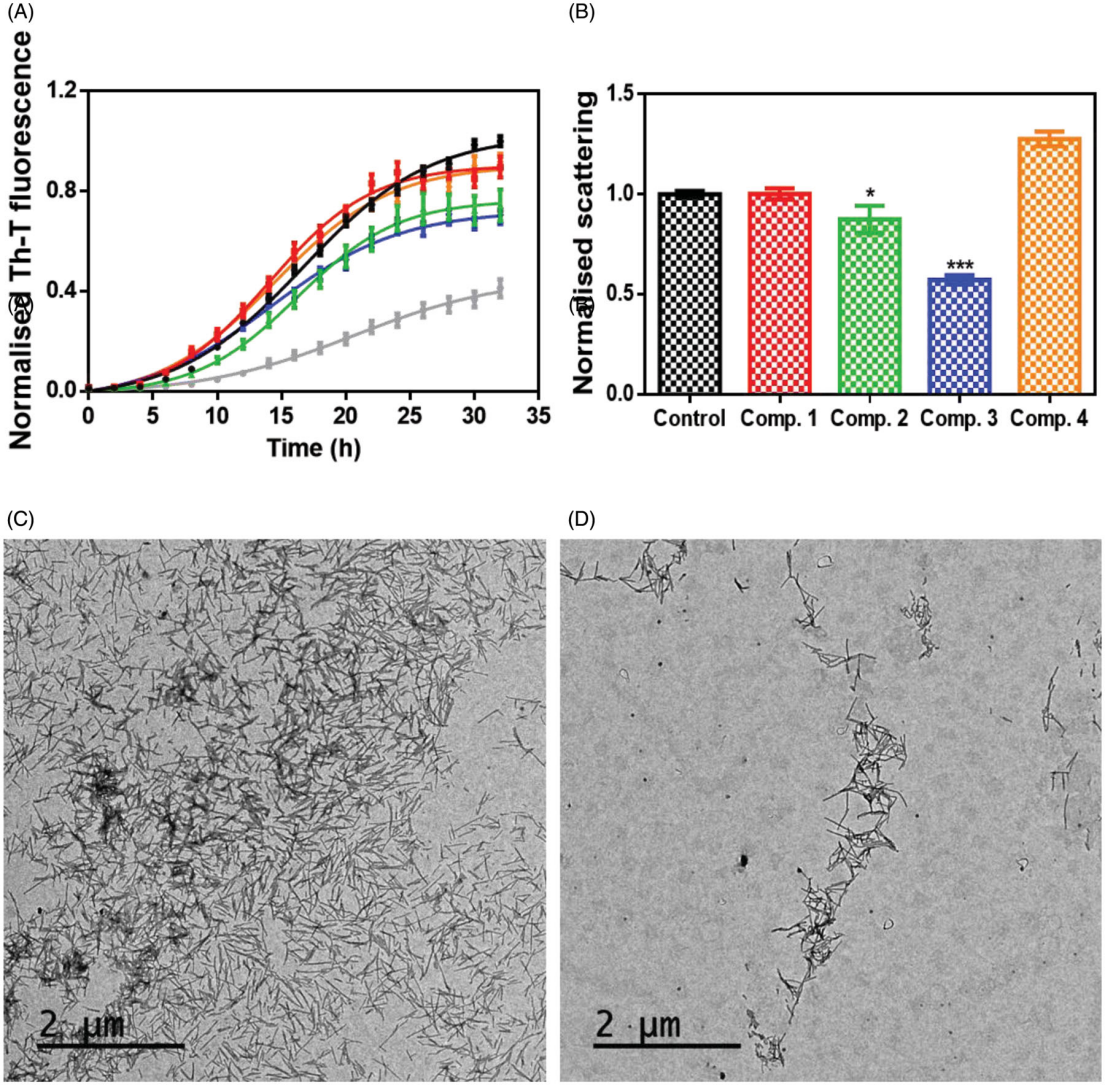
Inhibitory in vitro characterisation of compounds **1** to **4**. (A) Aggregation kinetic of α-syn in absence (black) and presence of 100 µM of Compound **1** (red), compound **2** (green), compound **3** (blue), compound **4** (orange) and SynuClean-D (grey), followed by Th-T fluorescence emission. (B) Light-scattering measurements at 300 nm in the absence (black) and presence of 100 µM of Compound **1** (red), compound **2** (green), compound **3** (blue) and compound **4** (orange). (C and D) Representative TEM images of untreated (C) and compound **3** treated samples (D). Th-T fluorescence is plotted as normalised means. Final points were obtained at 48 h. Error bars are represented as SE of mean values; ** *p* < 0.01 and *** *p* < 0.001.

The anti-aggregation potential of selected compounds (**1**–**4**) was evaluated by a robust protocol previously applied in the identification of strong inhibitors of α-syn aggregation^16^^,^^24^ like SynuClean-D, a well-known inhibitor of α-synuclein (α-Syn) aggregation^24^ that was used as reference compound in the same test. The presence of 100 µM of the compounds during the incubation of 70 µM of α-Syn, reduced Thioflavin-T (Th-T) fluorescence emission up to a 10, 26, 31 and 10% for compound **1**, compound **2**, compound **3** and compound **4**, respectively. As result, all tested compounds impacted the kinetic constants (see Figure 4(A)). Particularly, the compound **2** reduced the homogeneous nucleation rate constant (*k_b_* = 0.01591) when compared to untreated sample (k_b_ = 0.03234). In contrast, compound **3** reduced the autocatalytic rate constant (*k_a_* = 0.1892 h^−1^), compared to the control (*k_a_* = 0.2037 h^−1^). The compounds **1** and **4** did not impact significantly the aggregation rate constants. To confirm these results light-scattering measurements at 300 nm were performed at the end-point of the reaction, thus revealing a reduction of 13 and 43% of aggregates in the presence of compounds **2** and **3**, respectively.

To further assess the impact of the best candidate **3** on α-syn aggregation, end-point samples were analysed by transmission electron microscopy (TEM). Notably, TEM images certified a significant reduction in the amount of fibrillar material in the presence of compound **3** (Figure 4(D)), when compared to untreated samples (Figure 4(C)).

Therefore, this first screening highlighted that the best *in vitro* results were obtained for compound **3.** This compound is characterised by the presence of a pyridinyl-triazole moiety similarly to compound **2**, in which the nitrogen of the triazole ring presents a methyl substituent while an amino group is present in compound **3** in the same position. Considering the obtained results, we can speculate that the amino group could establish fundamental interaction with the protein important for the inhibition of α-syn aggregation.

### 3.2. Design and synthesis of new small molecules as derivatives of compound 3

**Scheme 1.**
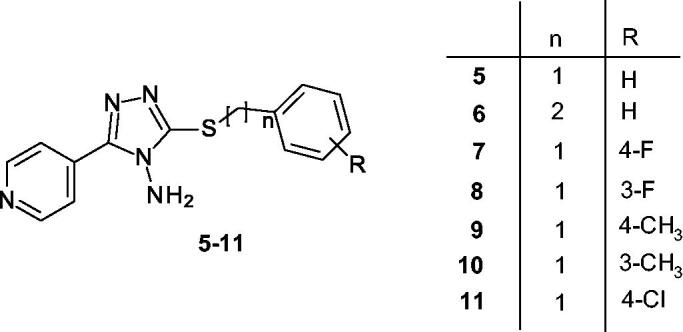
Designed pyridinyltriazole derivatives **5**–**11** inspired by compound **3**.

Based on the above reported results of biological screening, we chose compound **3** as promising lead compound for the rational design of a further series of pyridinyl-triazole derivatives **5–11** (Scheme 1) for which a very simple synthetic approach might be carried out.

**Scheme 2.**
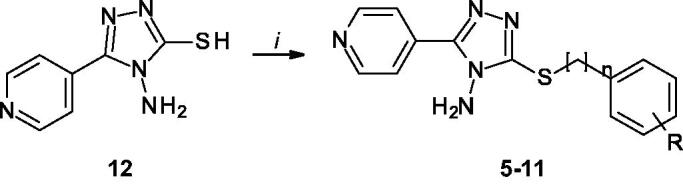
*Reagents and conditions*: *i*) Ar(CH_2_)_n_Br, NaOH, MeOH, rt.

In particular, we chose to maintain the pyridinyl-triazole moiety of inhibitor **3** and reduce the length of the linker between the sulphur atom and the phenyl ring. Moreover, we planned to extend the series by introducing selected substituents in meta and in para positions of phenyl ring. In detail, we introduced F, Cl or CH_3_ substituents in order to preliminary probe the influence of electron withdrawing group (EWG) or electron donating group (EDG) on the aggregation of α-syn. For each designed derivative we evaluated the adherence to the Lipinski’s rule and the absence of PAINS in silico by using SwissADME. Scheme 2 shows the synthetic procedure to obtain compounds **5-11** starting from the 4-amino-5–(4-pyridinyl)-4H-1,2,4-triazole-3-thiol **12**, that was coupled with the appropriate benzyl bromide in alkaline medium at room temperature.

### 3.3. Screening of the activity of the new derivatives 5–11

**Figure 5.**
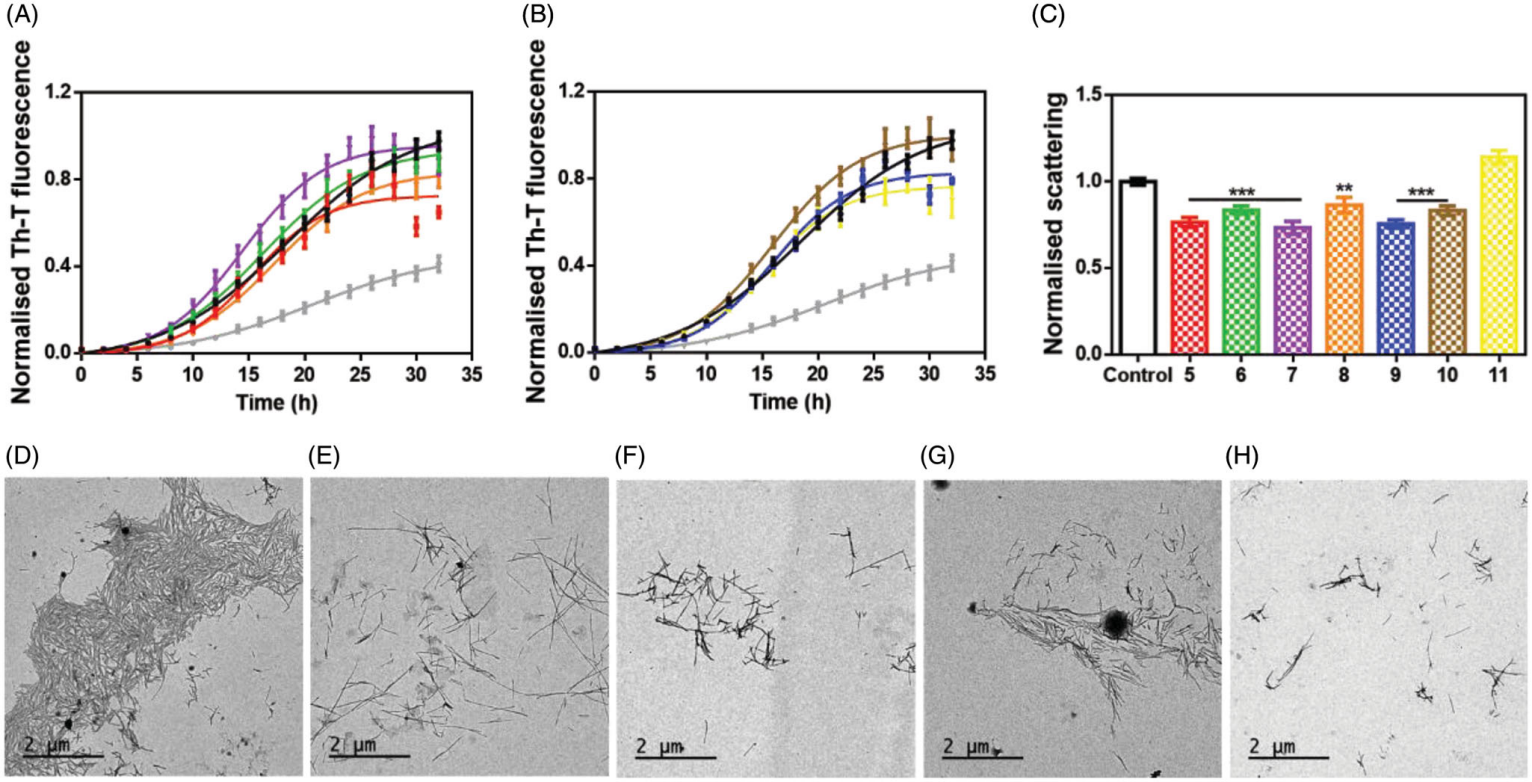
**Inhibitory in vitro characterisation of tested compounds 5–11.** (A and B) Aggregation kinetics of α-syn in the absence (black) and presence of 100 µM of compounds **5** (red), **6** (green), **7** (violet), **8** (orange), **9** (blue), **10** (brown), **11** (yellow) and SynuClean-D (grey), followed by Th-T fluorescence emission. (C) Light-scattering measurements at 300 nm in the absence (black) and presence of 100 µM of compounds **5** (red), **6** (green), **7** (violet), **8** (orange), **9** (blue), **10** (brown) and **11** (yellow). (D to G) Representative TEM images of untreated (D) and **5** (E), **8** (F), **9** (G) and **11** (H) treated samples. Th-T fluorescence is plotted as normalised means. Final points were obtained at 48 h. Error bars are represented as SE of mean values; ** *p* < 0.01 and *** *p* < 0.001.

The synthesised derivatives were tested in order to study their ability to reduce α-syn aggregation *in vitro* by using the method described above. Incubation of 70 µM α-Syn in the presence of 100 µM of the different compounds, followed by Th-T fluorescence measurements at the end of the reaction indicated that **5**, **8**, **9** and **11** could reduce the aggregation up to a 33, 15, 19 and 29%, respectively (Figure 5(A)). Light-scattering measurements at 300 nm indicated that **5**, **6**, **7**, **8**, **9** and **10** reduced the amount of aggregates up to a 23, 16, 26, 14, 24, 17%, respectively (Figure 5(B)). However, TEM analysis indicated that only **5** (Figure 5(E)), **8** (Figure 5(F)), **9** (Figure 5(G)) and **11** (Figure 5(H)) reduced the number of amyloid fibrils when compared to control samples (Figure 5(D)), in good agreement with the kinetic analysis.

### 3.4. Docking studies

In order to ascertain the binding mode of pyridinyl-triazole derivatives **3**, **5**, **8**, **9** and **11** that demonstrated the ability to reduce α-syn aggregation *in vitro*, we carried out molecular docking simulation by means of Autodock 4.2 suite;^29^ the computational studies were based on NMR structure of α-syn (PDB code 2N0A) as structural coordinates^30^.

**Figure 6.**
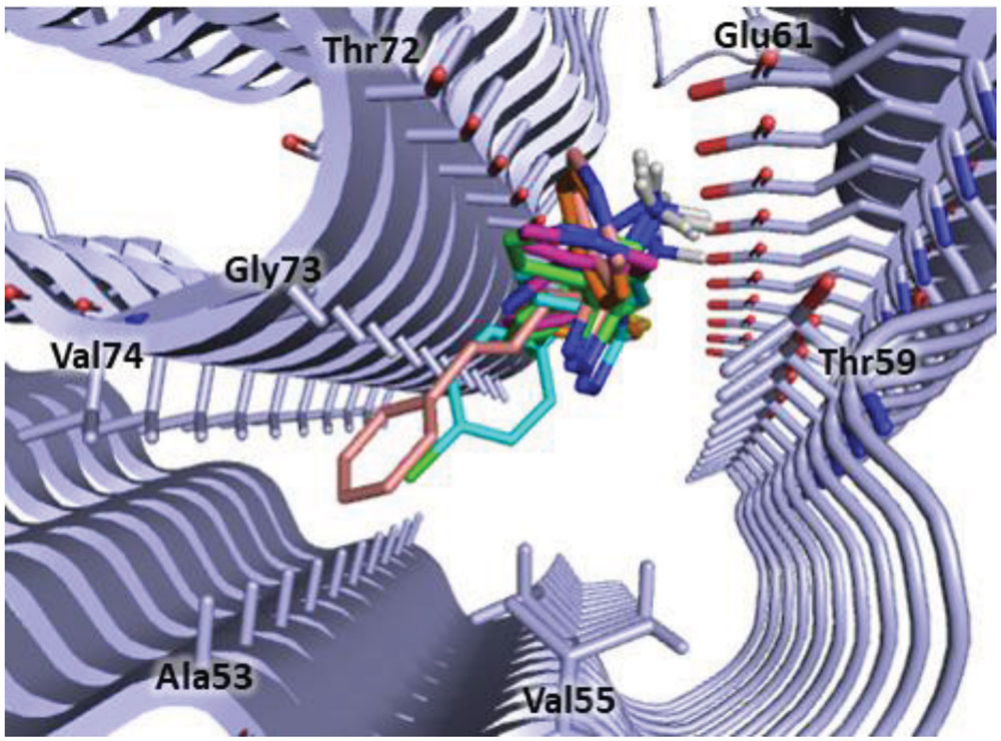
Binding site of α-syn identified through docking studies for this class of inhibitors. The image is created by PyMOL software (https://pymol.org).

Considering that in our above reported molecular modelling studies we selected polyphenolic and non-polyphenolic inhibitors (see Table 1) that bind the N-terminal region of α-syn, we hypothesised that also derivatives **3**, **5**, **8**, **9** and **11** could interact with same portion of the protein. For this reason, we defined this region as the search space for the docking simulation (see Material and Methods section). The docking results confirmed that all the tested compounds bind in the same site of α-syn located between the N-terminal and the NAC domains of the protein and lined by Ala53, Val55, Thr59, Glu61, Thr72, Gly73 and Val74 (Figure 6).

Notably, in a recent work, Pujols and co-workers identified the same portion of the protein as binding site of their inhibitor 2-hydroxy-5-nitro-6-(3-nitrophenyl)-4-(trifluoromethyl)nicotinonitrile through a induced-fit docking simulation^24^; therefore, these evidences further supported the hypothesis that this region could represent a binding site for small molecules on α-syn fibrils.

**Figure 7.**
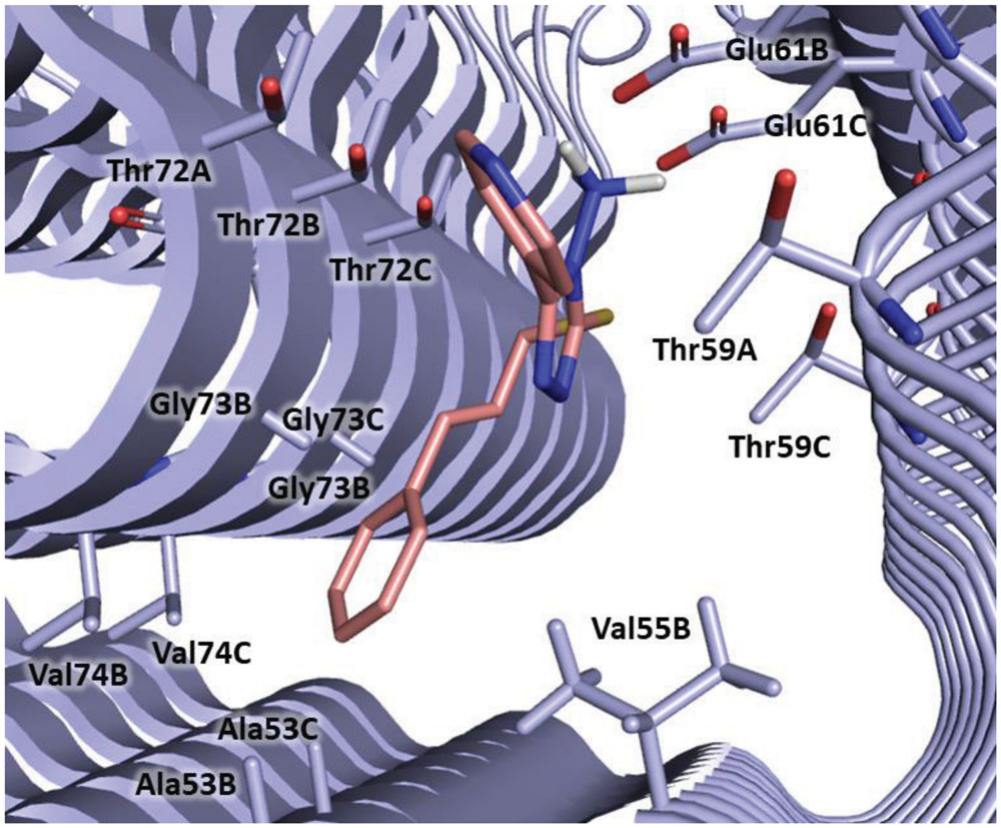
Plausible binding mode of compound **3** (pink stick). The interacting residues of the binding site are represented as light blues stick. The image is created by PyMOL software (https://pymol.org).

In detail, compound **3** could bind to α-syn by establishing a salt bridge between the amino group and Glu61 and π-anionic interaction between the pyridine ring and Glu61B. The cinnamyl moiety is oriented towards Ala53, Gly73 and Val74 and might engage hydrophobic interaction with Ala53. Furthermore, van der Waals interactions with Val55, Thr59, Thr72, Gly73 and Val74 could be observed (Figure 7).

**Figure 8.**
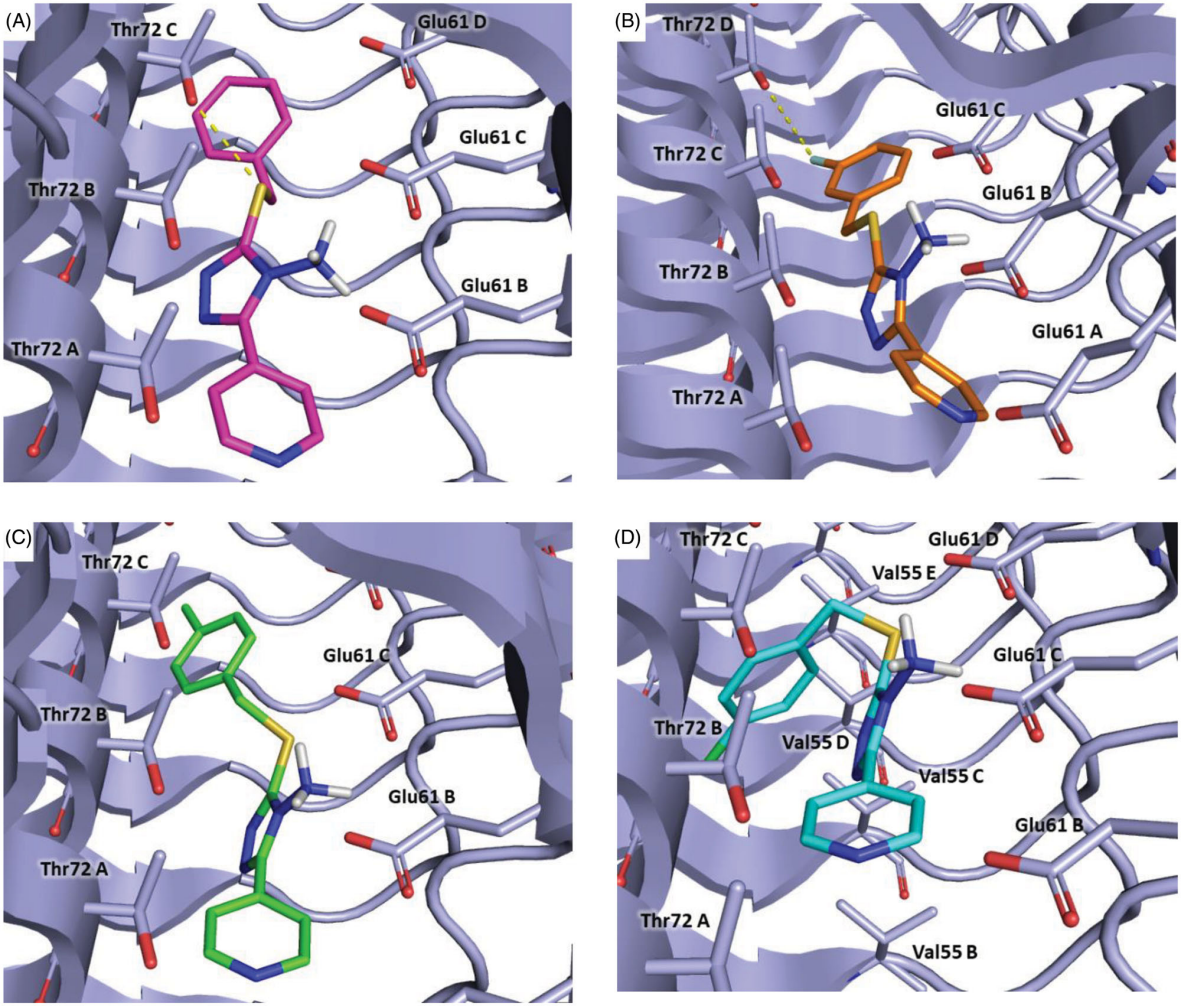
Plausible binding modes for compound **5** (magenta stick, panel A), **8** (orange stick, panel B), **9** (green stick, panel C) and **11** (cyan stick, panel D). The interacting residues of the binding site are represented as light blues stick. The images are created by PyMOL software (https://pymol.org).

In Figure 8, the binding mode of compounds **5**, **8**, **9** and **11** is displayed. The new derivatives showed a slight different binding orientation in comparison to the parent compound **3**, probably due to the shorter and more flexible linker between the sulphur atom and the aromatic ring.

In particular, all the inhibitors could interact with α-syn by a crucial salt bridge between the amino group and the side chain of Glu61. Moreover, for inhibitors **5**, **8**, **9** a pi-anion interaction was observed between the pyridine ring and Glu61B.

Additionally, compound **5** might form a hydrogen bond with Thr72C through the sulphur atom and pi-sigma interaction with Thr72C (Figure 8, Panel A). Concerning compound **8**, it might establish a halogen bond between the fluorine atom and Thr72D (Figure 8, Panel B). Instead, **9** could engage pi-sigma interaction with Thr72C (Figure 8, Panel C). Finally, compound **11** might establish van der Waals interactions with Val55C (Figure 8, Panel D).

## 4. Conclusion

The search for a cure of PD represents an important challenge in the pharmaceutical research field. The inhibition of α-syn aggregation has emerged as promising new therapeutic strategy for the treatment of PD. In this work, we described the generation of a ligand-based pharmacophore model, which was used as query to screen two chemical libraries. The hits selected from the virtual screening were tested *in vitro* to probe their ability to inhibit α-syn aggregation, resulting in the identification of the new hit **3**. Structural modifications were carried out on compound **3** obtaining four interesting derivatives. Finally, the binding mode of this new class of inhibitors on α-syn was investigated by molecular docking simulation. All the information gained from these studies could be useful for the design of novel inhibitors α-syn aggregation inhibitors bearing a pyridinyl-triazole scaffold.
